# Expression of CD47 in Endometrial Cancer and Its Clinicopathological Significance

**DOI:** 10.1155/2022/7188972

**Published:** 2022-03-04

**Authors:** Mei Yang, Chunfan Jiang, Lin Li, Hui Xing, Li Hong

**Affiliations:** ^1^Department of Obstetrics and Gynecology, Renmin Hospital of Wuhan University, Wuhan 430060, China; ^2^Department of Obstetrics and Gynecology, Xiangyang Central Hospital, Affiliated Hospital of Hubei University of Arts and Sciences, Xiangyang 441021, China; ^3^Department of Pathology, Xiangyang Central Hospital, Affiliated Hospital of Hubei University of Arts and Sciences, Xiangyang 441021, China

## Abstract

**Purpose:**

To study the prognostic value of CD47 in endometrial carcinoma (EC) and its correlation with clinicopathological variables.

**Methods:**

Next-generation sequencing data from The Cancer Genome Atlas was analyzed with the Kaplan–Meier curve, Cox's regression model, and ROC curve. A cohort of 544 specimens, including 344 cases of endometrial cancer, 92 cases of endometrial hyperplasia (EH), and 118 cases of normal endometrium (NE), were evaluated with immunohistochemistry and analyzed with statistical methods.

**Results:**

For TCGA data, CD47 expression in EC was considerably greater than in NE tissues. CD47 expression correlated significantly with age, clinical stage, histological grade, histological type, and menopause status. Kaplan–Meier analysis and Cox's regression model revealed that elevated CD47 expression was positively correlated with a poorer prognosis. ROC curve showed that CD47 had high specificity and sensitivity as an independent prognosis factor. In our cohort, CD47 expression was significantly stronger in EC than in NE. The strongly positive expression of CD47 could be observed in EC, but none was observed in NE. The CD47 expression rate ranked in descending order: atypical endometrium hyperplasia, complex endometrium hyperplasia, and simple endometrium hyperplasia. Atypical endometrium hyperplasia CD47 expression rate was much greater than either simple endometrium hyperplasia or complex endometrium hyperplasia. A substantial connection existed amongst CD47 expression and the clinical stage. Kaplan–Meier survival analysis demonstrated that CD47 expression was connected with overall survival (OS). Univariate analysis instead of the multivariate analysis revealed that CD47 expression was associated significantly with prognosis.

**Conclusions:**

CD47 is a critical part of the progress of pathogenesis in EC. CD47 expression correlates with multiple clinicopathological variables and is a potential prognostic risk factor.

## 1. Introduction

Endometrial cancer (EC) is one of the most common female reproductive system malignant tumors with distinct biological behavior. About 634 per 100,000 newly diagnosed cases and 21.8 per 100,000 mortalities in 2017 were claimed by this disease in China [1].

EC is traditionally categorized into two histological types. Type I EC (endometrioid carcinoma) is the most common and is related to estrogen excess, and this type generally belongs to low grade. Type II EC consisting of serous carcinoma, clear cell carcinoma, and others is much more likely to be high grade [2]. EC can be early diagnosed by fractional curettage or endometrial biopsy with the onset symptom of postmenopausal vaginal bleeding. However, about 30% of advanced stage endometrial cancer diagnosed patients do not improve long-term survival even with recommended treatment strategy including surgery, platinum-based chemotherapy, and radiotherapy [3, 4]. The application of CA-125 in clinical practice may be useful in advanced stages and serous carcinoma [5]; however, there is no biomarker with special sensitivity and specificity to predict prognosis and therapeutic effects. Thus, exploring the pathological mechanism and looking for desirable biomarkers to increase the rate of early diagnosis of EC are the key to ameliorate prognosis.

CD47 is a transmembrane immune-regulatory protein expressed on a variety of cell membranes [6]. This molecule has a ligand to interact with signal regulatory protein alpha (SIRP*α*) on neutrophils, dendritic cells, macrophages, and T and B lymphocytes to activate various cellular metabolisms, such as nitric oxide, calcium homeostasis, and hydrogen sulfide biosynthesis [7]. The overexpression of CD47 on the aged and superfluous cells initiates the phagocytosis by macrophages to keep the vitality of a healthy cell population. In ovarian carcinoma, breast carcinoma, melanoma, and gastric carcinoma, overexpression of CD47 was found to correlate with poor survival [8–12]. On the contrary, inhibiting CD47 expression could increase the ability of macrophages to eradicate different types of cancer [13]. These exciting experiment results make CD47 a promising new biomarker for cancer therapy and prognosis assessment. Although CD47 has been involved in cancer progression, at the cell level in vitro CD47 was discovered to be a critical part in enhancing cell migration ability, viability, and inhibiting apoptosis in endometrial carcinoma cells via the PI3K_Akt_mTOR Signaling Pathway [14]. Its prognostic value and its correlation with clinicopathological variables in large numbers of endometrial cancer stay vague.

This study explored CD47 expression and available clinical variables from The Cancer Genome Atlas (TCGA) data and also detected CD47 expression in EC, EH, and NE with our collected samples to provide a theoretical basis for screening a potential biomarker to evaluate prognosis and promote new drug development.

## 2. Materials and Methods

### 2.1. TCGA Data Analyses

RNAseq data including 552 ECs and 35 adjacent cancer tissues in HTSeq-FPKM (Fragments Per Kilobase Per Million) format and the clinical records were downloaded from TCGA. Some items of the clinical records were incomplete. The RNAseq data in the FPKM format was adapted to the TPM (transcripts per million reads) format and conducted with Log2 conversion before bioinformatics analysis. Preprocession and bioinformatics analysis of the downloaded raw data were conducted using the *R* 3.6.3 software.

### 2.2. Ethics Approval

The research was conducted following the Helsinki Declaration as well as given approval from the Ethics Committee of Xiangyang Central Hospital (reference number 2021005). Medical data were completely anonymized. Investigators were blinded for all the clinical information during the analyses.

### 2.3. Case Selection

A group of 544 formalin-fixation paraffin-embedded specimens, containing 344 EC cases, 92 endometrial hyperplasia cases, and 118 normal endometrium cases, were obtained from Xiangyang Central Hospital, Hubei province, from 2006 to 2011 with needed clinical data. All the patients were Han People. The age of endometrial cancer patients was between 29 and 83 years, mean ± SD (55.05 ± 8.70) years; endometrial hyperplasia patients between 29 and 68 years, mean ± SD (45.80 ± 6.22) years; normal endometrium participants between 24 and 57 years, mean ± SD (39.86 ± 8.32) years. EC information on clinicopathological variables was listed in Table 1. EH included 57 cases of simple hyperplasia, 15 cases of complex hyperplasia, and 20 cases of atypical hyperplasia. NE comprised 83 cases of proliferative phase and 35 cases of secretory phase. Two senior pathologists examined all cases individually for a second time to guarantee the diagnosis precision. For EC cases, tissue blocks with abundant carcinoma and adjacent normal endometria were selected.

### 2.4. Immunohistochemistry

The Ventana Benchmark ULTRA automated staining system (Ventana Medical Systems, Tucson, AZ) CD47 was utilized for immunohistochemical staining implemented with 3 µm thick sections following manufacturer protocol. Mouse monoclonal anti-CD47 antibody (Clone No. 12730, 1 : 100 dilution, Santa Cruz) was the primary antibody used. The reaction was visualized with 3,3′-diaminobenzidine (DAB). Determined EC cases with CD47 strong expression were selected as the positive control. PBS was applied as a negative control in place of the primary antibody. Pictures were scanned with TEKsqray Digital Slide Scanner (Shengqiang Tech Ltd., Shenzhen, China).

### 2.5. Immunohistochemical Scoring

Positive immunostainings were located on membranes and cytoplasm of cancer cells, and any immunostainings in endothelial, lymphocytic, or desmoplastic tissue were discounted. Scoring of CD47 immunostaining was completed as earlier explained using minor modification [15]. Positive cell intensity and percentage were obtained by counting cancer cells under 400 × magnification from 10 randomly chosen visual fields. The intensity of immunostaining was scored 0, 1, 2, 3, accounting for no signal, weak signal (light yellow), moderate signal (yellowish-brown), and strong signal (brown), individually. Scores of 0–4 for positive cell percentage were 0=<5%, 1 = 5%–25%, 2 = 21%–50%, 3 = 51%–75%, and 4=>75%. The final results for a single case were established by the sum of the two scores: negative (−) 0 or 1 sum, weakly positive (+) 2 or 3 sum, moderately positive (++) 4 or 5 sum, and strongly positive (+++) greater than 6 sum. Two senior pathologists assessed all sections independently. The results were judged by a third pathologist once a disagreement occurred.

### 2.6. Data Statistics

The correlation amongst CD47 expression and clinicopathological variables was tested by chi-square tests and Fisher exact test. The Kaplan–Meier curve presented the survival probability and was analyzed using the log-rank test. Cox's regression model identified the prognostic risk factors. The specificity and sensitivity of CD47 as an independent prognostic factor were evaluated with the performance of the ROC curve analysis. *P* < 0.05 was deemed statistically significant. Statistical calculation was done using the SPSS 23.0 software (IBM, Chicago, IL, USA).

## 3. Results

### 3.1. Upregulation of CD47 Expression in EC Using TCGA Data

**Figure 1:**
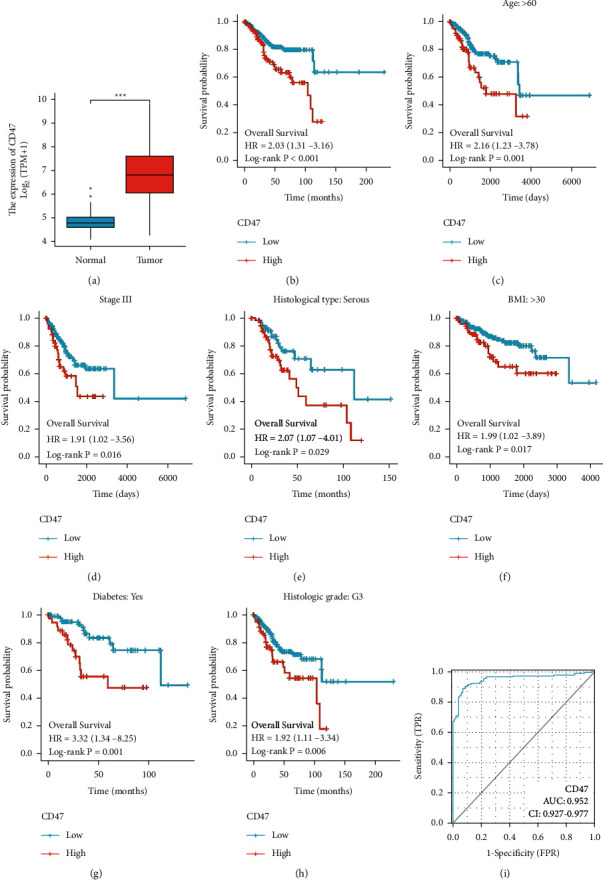
Analysis of CD47 expression and prognosis in EC using TGCA data. (a) CD47 expression differences between NE and EC are shown in the box-and-whisker plot. (b) Overall survival curve. Subgroup survival curve according to age >60: (c) stage III, (d) histological type: serous, (e) BMI >30, (f) diabetes, (g) histological grade: G3, and (h) ROC curve according to CD47 expression (i).

To explore CD47 expression levels in EC, we analyzed TPM values with Log2 conversion. The expression of CD47 was higher than that of normal endometrial tissues (*p* < 0.001, Figure 1(a)).

### 3.2. Correlation between CD47 Expression and Clinicopathological Variables Using TCGA Data

CD47 expression correlated significantly with histological grade (*p* < 0.001), histological type (*p* < 0.001), age (≤60 vs. >60, *p* < 0.001), clinical stages (stages I + II vs. stages III + IV, *p* = 0.024), and menopause status (*p*=0.002, Table 2). There were no significant differences in weight, height, BMI, tumor invasion, and hormones therapy between the low and high CD47 expression group (*p* > 0.05, Table 2).

### 3.3. The Prognostic Value of CD47 Expression in EC Using TCGA Data

Kaplan–Meier analysis revealed that high CD47 expression was positively correlated with a poorer prognosis (HR = 2.03, CI = 1.31–3.16, *p* < 0.001, Figure 1(b)). Subgroup OS analysis pointed out that patients >60, stages III + IV, serous carcinoma, BMI >30, diabetes, and histological grade G3 combined with the high CD47 expression had a poorer prognosis when weighed against those with the low CD47 expression (*p* < 0.01, Figures 1(c)–(h)).

Univariate regression analysis showed that clinical stage (stages I and II vs. stages III and IV), age (>60 vs. ≤60), histological type (mixed and serous vs. endometrioid), histologic grade (G3 vs. G1 and G2), tumor invasion (≥50 vs. <50), and CD47 (low vs. high) were the factors influencing OS (*p* < 0.05). Multivariate regression analysis revealed that the clinical stage and CD47 were the independent risk factors for EC progression (*p* < 0.05, Table 3).

A ROC curve showed that CD47 could be chosen as a biomarker to foresee EC prognosis with high specificity and sensitivity (AUC = 0.952, CI = 0.927–0.977, Figure 1(i)).

### 3.4. CD47 Expression in Normal Endometrium and Endometrial Hyperplasia and Endometrial Carcinoma

**Figure 2:**
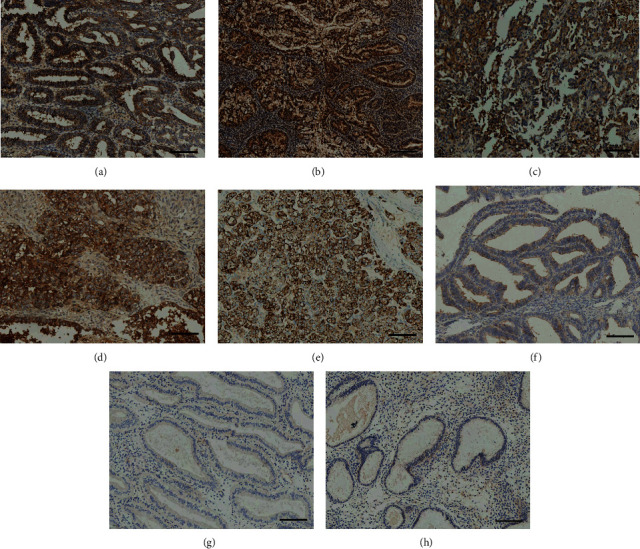
Representative illustrations of the CD47 expression in EC, EH, and NE with immunohistochemistry. (a) G1 endometrioid carcinoma. (b) G2 endometrioid carcinoma. (c) G3 endometrioid carcinoma. (d) Serous carcinoma. (e) Clear cell carcinoma. (f) Atypical endometrial hyperplasia. (g) Proliferation phase endometrium. (h) Secretory phase endometrium. 100X pictures were presented. Scale bars, 300 *μ*m.

Two CD47 expression statuses including positivity and strong positivity were set to look for a suitable cut-off value to distinguish a significant difference between groups. CD47 positive and strongly positive expression rates in the endometrial cancer group were considerably greater than the endometrial hyperplasia group and the normal endometrium group (*P* < 0.01). A declining trend of CD47 positive and strongly positive expression rate existed from EC to EH and then to NE. Comparing between the simple hyperplasia group and the complex hyperplasia group, CD47 positive and strongly positive expression rates did not show significant differences; however, comparing between either of the two groups and the atypical hyperplasia group, the positive and strongly positive expression rates showed significant differences (*P* < 0.01). CD47 positive and strongly positive expression did not show significant differences between the proliferation phase group and the secretory phase group (*P* > 0.05, Table 4). Examples of CD47 immunostainings in EC, EH, and NE were represented in Figure 2.

### 3.5. Relationship between CD47 Expression and Clinicopathological Variables of Endometrial Cancer

CD47 positive and the strongly positive expression rates did not show significant differences in EC types, histological types, histological grade, and Ki67 expression. Significant differences existed among different clinical stages (*P* < 0.01). For infiltration depth, lymph node metastasis, and P53 expression, CD47 positive and strong positive expression rates revealed different results. For CD47 strongly positive expression rates, a significant difference existed among infiltration depth and lymph node metastasis groups; however, there was no significant difference among these two groups for the positive expression rate of CD47 (*P* < 0.01). Comparing between P53 wild-type and mutant type, the CD47 positive expression rate was significantly different (*P* < 0.01); however, there was no significant variation for the strongly positive CD47 expression rate (Table 5).

### 3.6. Prognosis Analysis

**Figure 3:**
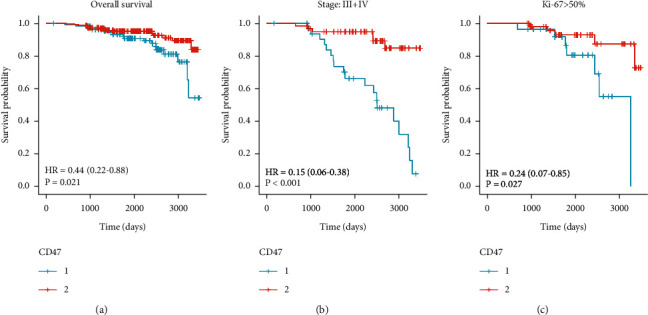
Kaplan–Meier survival analysis of EC patients with various CD47 expression levels. (a) Overall survival curve. (b) Subgroup survival according to stages III + IV. (c) Ki67 > 50%.

Kaplan–Meier survival analysis demonstrated that endometrial cancer patients with positive CD47 expression had considerably greater mortality than those with no positive CD47 expression (*P* < 0.01). Subgroup Kaplan–Meier survival analysis demonstrated that, in stages III-IV and Ki67 > 50% groups, high CD47 expression correlated significantly with a poorer prognosis (*P* < 0.01, Figure 3).

Univariate analysis showed that age, CD47 expression, clinical stage, histological grade, infiltration, lymph node metastasis, and Ki67 index correlated significantly with prognosis (*P* < 0.05). However, only the clinical stage and lymph node metastasis show a significant correlation with prognosis by multivariate analysis (*P* < 0.05, Table 6).

## 4. Discussion

The immune checkpoint is a type of costimulatory and inhibitory molecule responsible for antigen recognition regulation of T cell receptors (TCR) in the immune response process [16]. By dysregulating immune checkpoint-related proteins, cancer cells are able to easily escape immune attacks. Immune checkpoint inhibitors block immune checkpoint-related proteins from binding with their partners and thereby allow the T-cells to kill cancer cells [17]. Nowadays, the most successful example is the development of anti-PD-1/PD-L1 antibodies. Durvalumab, atezolizumab, nivolumab, and pembrolizumab were put into a clinic in succession, and many patients get to benefit from them with gastric cancer, lung cancer, and esophagus cancer [18]. Yet, not all PD-L1-positive cancers react to anti-PD-1/PD-L1 antibodies, and side effects such as rash, diarrhea, and colitis occur in some patients. The success of anti-PD-1/PD-L1 antibody development and its drawback make immune checkpoint a most potential research area, and scientists are still on the hunt for a new target.

CD47 like PD-L1 is widely overexpressed on the membrane of many solid tumors, including triple-negative breast cancer, ovary cancer, bladder cancer, gastric cancer, and so on [8–10, 12, 19]. CD47 overexpression is a useful strategy for solid tumors to escape from immune attack by delivering a “do not eat me” signal to avoid phagocytosis via the binding of SIRP*α* expressed on phagocytes [6, 7]. Currently, Hu5F9-G4, a humanized anti-CD47 antibody, is undergoing evaluation in a phase I trial, and it was reported that Hu5F9-G4 could significantly suppress the progression of advanced solid malignancies [20]. It brings a new hope for doctors and patients to treat solid malignancies. Advanced EC patients also need a new medicine in their drug box, and thereby, evaluating the prognostic value of CD47 expression on EC appears very important.

In this study, we first analyzed the TCGA data and discovered that CD47 expression in EC was drastically greater than in normal endometrial tissues. Additional analysis of the relationship amongst CD47 expression and clinicopathological variables demonstrated that CD47 expression correlated significantly with age, clinical stage, histological type, histological grade, and menopause status. Kaplan–Meier analysis revealed that high CD47 expression was positively correlated with a poorer prognosis. Subgroup OS analysis showed that age >60, stages III + IV, serous carcinoma, BMI >30, diabetes, and histological grade G3 combined with the high CD47 expression had a poorer prognosis. Univariate and multivariate analysis indicated that only the clinical stage and CD47 could be the independent risk factors to evaluate the prognosis of EC. A ROC curve showed that CD47 had high specificity and sensitivity as an independent prognosis factor.

Sercan et al. stated that CD47 expression was considerably higher in EC and was associated with histologic grade [21]. However, CD47 expression was not in association with OS and other clinicopathological variables. Sercan's conclusions do not exactly coincide with our observation with TCGA data.

We next analyzed the CD47 expression in our collected cohort. It was discovered that the CD47 expression is considerably stronger in endometrial cancer than in normal endometrium. The strongly positive expression of CD47 could be observed in endometrial cancer, but none was observed in normal endometrium. We also explored the CD47 expression in endometrial hyperplasia. Endometrial hyperplasia could be divided into simple endometrium hyperplasia, complex endometrium hyperplasia, and atypical endometrium hyperplasia. Atypical endometrium hyperplasia is a precancerous lesion. The CD47 expression ranks in descending order, atypical endometrium hyperplasia > complex endometrium hyperplasia > simple endometrium hyperplasia. No significant difference existed between simple endometrial hyperplasia and complex endometrial hyperplasia; however, the atypical endometrium hyperplasia CD47 expression rate was much greater compared to either simple endometrium hyperplasia or complex endometrium hyperplasia. The CD47 expression in precancerous lesions was barely reported in published literature, and our results support that CD47 possibly takes part in oncogenesis and has a critical position in the progression from normal, hyperplasia, and atypia then to EC.

By analyzing our cohort, a significant correlation existed between CD47 expression and the clinical stage. However, the establishment of a significant correlation between infiltration, lymph node metastasis, P53, and CD47 expression depended on the choice of cut-off value (positive or strongly positive). Kaplan–Meier survival analysis demonstrated that CD47 expression was linked with OS. Subgroup Kaplan–Meier survival analysis demonstrated that stages III-IV and Ki67 > 50%, combined with high CD47 expression correlated significantly with a poorer prognosis. Univariate analysis showed that age, CD47 expression, clinical stage, histological grade, infiltration, lymph node metastasis, and Ki67 index correlated significantly with prognosis. However, only the clinical stage and lymph node metastasis show a significant correlation with prognosis by multivariate analysis.

Comparing the results from TCGA data with our cohort, the clinical stage is the most stable prognosticator compared to others with different statistical methods. Routine clinical practices have demonstrated that the clinical stage is a scientific and reasonable prognosticator with overall consideration of infiltration depth, lymph node invasion, and distal metastasis. CD47 showed a significantly different expression in descending order from normal and atypia then to EC. Survival analysis discovered that high CD47 expression had a poorer prognosis. CD47 expression had a significant correlation with several clinicopathological variables especially the clinical stage. Cox's regression model also showed that CD47 had an important prognosis value for EC except for multivariable analysis with our cohort. More serious carcinoma and cases with rare pathological types were included in TCGA data, and to ensure the integrity of clinical data, some cases with incomplete information were not incorporated in our cohort. These data differences may account for the reasons of nonstatistical significance in multivariable analysis with our cohort.

Several studies had reported that the CD47 overexpression is associated with poor prognosis and clinicopathological variables in different cancer patients [8, 9, 11]. Cell and animal studies revealed that downregulation of CD47 significantly suppressed the proliferation and metastasis of cancer cell lines and caused tumor reduction in heterotopic and orthotopic xenograft mouse models [22]. Further researches on molecular mechanisms showed that activated STAT3 pathway by IL-6 upregulated CD47 expression in hepatocellular carcinoma cell lines, and the IL-6-STAT3 axis blockage reduced cancer cells' antiphagocytic ability via downregulation of CD47 expression [23]. Literature of molecular mechanisms on the pathogenesis of EC is rare. PI3K/Akt/mTOR signaling pathway activation via upregulation of CD47 expression enhances cellular viability and migration ability but suppresses endometrial carcinoma cell apoptosis [14]. Blocking the CD47-SIRPa interaction promotes phagocytosis of polarized-M2 macrophages to suppress tumor progression [24]. These experiment results demonstrated that CD47 could be a critical part of the progress of pathogenesis in EC.

Several drawbacks existed in our study. In our cohort, multivariable Cox's regression did not show CD47 correlated with prognosis. We have explored the possible reasons for nonstatistical significance. Multiple clinicopathological variables influence the relationship between CD47 and prognosis. More specimens particularly with rare pathological types should be brought into our study and maybe achieve a possible positive result. A standard cut-off like PD-L1 and HER-2 is important to evaluate immunohistochemistry and predict the relationship between biomarkers and clinicopathological variables [25, 26]. Multiple studies have put forward different cut-offs to evaluate CD47 expression, which brings inconsistent observation results [12, 27, 28]. Further studies need to be performed to validate the practicability of these cut-offs. Clinical data shows that CD47 is an important molecule in the pathogenesis of EC; however, complex molecular mechanism is still unclear. Future studies to determine CD47 details on molecular oncogenesis of endometrial carcinoma are warranted.

## 5. Conclusion

In conclusion, CD47 could be a critical part of the progress of pathogenesis in EC. CD47 expression correlates with multiple clinicopathological variables and is a potential prognostic risk factor.

## Figures and Tables

**Table 1:** Baseline characteristics of EC patients.

Characteristics	Levels	N (%)
n		344
Age, mean ± SD	29–83	55.05 ± 8.70
Histological type	I	325 (94.5%)
	II	19 (5.5%)
Clinical stage	1	247 (71.8%)
	2	62 (18%)
	3	33 (9.6%)
	4	2 (0.6%)
Histological grade	G1	190 (55.2%)
	G2	96 (27.9%)
	G3	58 (16.9%)
Infiltration depth	<50%	270 (78.5%)
	>50%	74 (21.5%)
P53	Mutant	79 (23%)
	Wild	265 (77%)
Ki67	<50%	268 (77.9%)
	>50%	76 (22.1%)
Lymph node metastasis	Yes	31 (9.0%)
	No	184 (53.5%)
	No lymph node cleaning	129 (37.5%)

**Table 2:** CD47 expression in endometrial cancer with different clinicopathological variables using TCGA dada.

Characteristics	Low expression of CD47 (%)	High expression of CD47 (%)	*P* value
n	276	276	
Age, mean ± SD	62.04 ± 11.79	66.12 ± 9.9	
Clinical stage			0.024
Stages I + II	209 (37.8%)	184 (33.3%)	
Stages III + IV	57 (12.1%)	92 (16.7%)	
Age			<0.001
≤60	126 (23%)	80 (14.6%)	
>60	149 (27.1%)	194 (35.3%)	
Weight (Kg)			0.057
≤80	111 (21%)	132 (25%)	
>80	155 (29.4%)	130 (24.6%)	
Height (cm)			0.364
≤160	119 (22.8%)	128 (24.5%)	
>160	145 (27.7%)	131 (25%)	
BMI			0.730
≤30	105 (20.2%)	107 (20.6%)	
>30	158 (30.4%)	149 (28.7%)	
Histological type			<0.001
Endometrioid	245 (44.4%)	165 (29.9%)	
Mixed	9 (1.6%)	15 (2.7%)	
Serous	22 (4%)	96 (17.4%)	
Histologic grade			<0.001
G1	60 (11.1%)	38 (7%)	
G2	73 (13.5%)	47 (8.7%)	
G3	141 (26.1%)	182 (33.6%)	
Tumor invasion (%)			0.882
<50	139 (29.3%)	120 (25.3%)	
≥50	113 (23.8%)	102 (21.5%)	
Menopause status			0.002
Pre	27 (5.3%)	8 (1.6%)	
Peri	11 (2.2%)	6 (1.2%)	
Post	216 (42.7%)	238 (47%)	
Hormones therapy			1.000
No	148 (43%)	149 (43.3%)	
Yes	23 (6.7%)	24 (7%)	

**Table 3:** Univariate and multivariate analysis of the prognosticators of EC using TCGA data.

Characteristics	Total (n)	Univariate analysis		Multivariate analysis	
		HR (95% CI)	*P* value	HR (95% CI)	*P* value
Clinical stage (stages I and II vs. stages III and IV)	542	3.270 (2.145–4.984)	<0.001	2.671 (1.266–5.637)	<0.001
BMI (>30 vs. ≤30)	519	0.967 (0.636–1.470)	0.876		
Age (>60 vs. ≤60)	549	1.847 (1.160–2.940)	<0.010	1.301 (0.625–2.711)	0.482
Weight (>80 vs. ≤80)	528	1.060 (0.699–1.607)	0.784		
Height (>160 vs. ≤160)	523	1.153 (0.758–1.753)	0.507		
Histological type (mixed and serous vs. endometrioid)	552	2.628 (1.746–3.957)	<0.001	1.620 (0.772–3.402)	0.202
Histologic grade (G3 vs. G1 and G2)	541	3.281 (1.907–5.643)	<0.001	1.344 (0.618–2.923)	0.455
Tumor invasion (%) (≥50 vs. <50)	474	2.813 (1.744–4.535)	<0.001	1.222 (0.603–2.475)	0.578
Menopause status (post vs. pre and peri)	506	1.050 (0.507–2.175)	0.895		
Diabetes (yes vs. no)	451	1.172 (0.731–1.878)	0.510		
Hormones therapy (yes vs. no)	344	0.801 (0.380–1.689)	0.560		
CD47 (low vs. high)	552	1.021 (1.010–1.032)	0.015	1.018 (1.007–1.029)	0.021

**Table 4:** CD47 expression in normal endometrium, endometrial cancer, and endometrial hyperplasia.

Tissue type	Case	-	+	++	+++	Positive cases	Positive rate (%)	Strong positive cases	Strong positive rate (%)
Endometrial cancer	344	85	143	85	31	259	75.29^*∗*^	116	33.72 *∗*
Endometrial hyperplasia	92	65	23	4	0	27	29.34	4	4.34
Simple hyperplasia	57	49	8	0	0	8	14.04^*∗∗*^	0	0
Complex hyperplasia	15	11	4	0	0	4	26.67	0	0
Atypical hyperplasia	20	5	11	4	0	15	75.00	4	20.00
Normal endometrium	118	89	29	0	0	29	24.58	0	0
Proliferative phase	83	66	17	0	0	17	20.48	0	0
Secretory phase	35	23	12	0	0	12	34.29	0	0

Note. ^*∗*^ weighed against endometrial hyperplasia group and normal endometrium group, *P* < 0.01; ^*∗∗*^ weighed against the atypical hyperplasia group, *P* < 0.01.

**Table 5:** CD47 expression in endometrial cancer with different clinicopathological variables.

Features		Cases	Positive cases	Positive rate (%)	Strong positive cases	Strong positive rate (%)	*P* value (positive)	*P* value (strong positive)
EC types	I	321	244	76.01	106	33.02	0.315	0.362
	II	23	15	65.22	10	43.48		
Histological type	Endometrial adenocarcinoma	321	244	76.01	106	33.02	0.335	0.46
	Serous carcinoma	14	10	71.43	7	50.00		
	Clear cell carcinoma	9	5	55.56	3	33.33		
Infiltration depth	<1/2 muscle layer	270	197	72.96	62	22.96	0.068	<0.01
	>1/2 muscle layer	74	62	83.78	36	48.65		
Clinical stage	I	247	171	69.23	51	20.65	<0.01	<0.01
	II	62	58	93.55	39	62.90		
	III + IV	35	30	85.71	26	74.29		
Histological grade	G1	190	146	76.84	60	31.58	0.714	0.265
	G2	96	71	73.96	31	32.29		
	G3	58	42	72.41	25	43.10		
Lymph node metastasis	No	184	134	72.83	50	27.17	0.406	<0.01
	Yes	31	26	83.87	20	64.51		
	No lymph node cleaning	129	99	76.74	46	35.66		
P53	Mutant	79	49	62.03	24	30.38	0.03	0.501
	Wild type	265	210	79.25	92	34.72		
Ki67 proliferation index	>50%	76	56	73.68	27	35.53	0.763	0.784
	<50%	268	203	75.75	89	33.20		

**Table 6:** Screening of prognosticators in EC patients with univariate and multivariate analysis.

Characteristics	n	HR (95% CI) univariate analysis	*P* value univariate analysis	HR (95% CI) multivariate analysis	*P* value multivariate analysis
Age	344	1.066 (1.026–1.108)	<0.001	1.031 (0.984–1.080)	0.201
CD47 (positive vs. negative)	344	0.438 (0.218–0.881)	0.021	0.754 (0.349–1.630)	0.473
Cancer type (I vs. II)	344	10.275 (5.011–21.070)	<0.001	1.928 (0.629–5.907)	0.251
Clinical stage (I and II vs. III and IV)	344	3.907 (2.714–5.625)	<0.001	2.410 (1.280–4.535)	0.006
Histological grade (G3 vs. G1 and G2)	344	2.491 (1.756–3.532)	<0.001	0.861 (0.463–1.601)	0.636
Infiltration depth (<1/2 vs. >1/2)	344	4.533 (2.250–9.131)	<0.001	0.432 (0.129–1.438)	0.171
P53 (mutant vs. wild type)	344	0.777 (0.349–1.733)	0.538		
Ki67 (>50% vs. <50%)	344	2.244 (1.094–4.603)	0.028	1.218 (0.545–2.719)	0.631
Lymph node metastasis (yes vs. no)	344	13.845 (6.872–27.894)	<0.001	4.675 (1.358–16.096)	0.015

## Data Availability

The data are available on request to the authors.
